# Supplementary material for: Peptide-binding specificity prediction using fine-tuned protein structure prediction networks
Source: Proc Natl Acad Sci U S A. 2023 Feb 21;120(9):e2216697120. doi: 10.1073/pnas.2216697120 (PMC9992841; doi:10.1073/pnas.2216697120)
Supplement: Supplementary file 1 — Appendix 01 (PDF) [file pnas.2216697120.sapp.pdf]

# Supporting Information for
Peptide binding specificity prediction using fine-tuned protein structure prediction networks

Amir Motmaen[1,2,3], Justas Dauparas[1,2], Minkyung Baek[1,2], Mohamad H. Abedi[1,2,4], David Baker[1,2,4*], Philip Bradley[2,5*]

[1] Department of Biochemistry, University of Washington, Seattle, WA, USA.
[2] Institute for Protein Design, University of Washington, Seattle, WA, USA.
[3] Bioengineering Graduate Graduate Program, University of Washington, Seattle, WA, USA.
[4] Howard Hughes Medical Institute, University of Washington, Seattle, WA, USA.
[5] Division of Public Health Sciences, Fred Hutchinson Cancer Research Center, Seattle, WA, USA.

* To whom correspondence should be addressed: David Baker, Philip Bradley

**Email:** dabaker@uw.edu, pbradley@fredhutch.org

**This PDF file includes:**

Supporting text
Figures S1 to S11
Tables S1 to S2
SI References

**Supplementary discussion on model training**

The combined structure prediction-classification model adds two additional "logistic regression" parameters to the internal AlphaFold model parameters. These parameters, a slope and a switch-point, transform the mean MHC:peptide residue-residue confidence measure (the 'PAE' score) into a binder/non-binder probability that ranges from 0 to 1. A central hypothesis of this study was that fine-tuning the internal AlphaFold parameters in the context of binder classification would lead to significant improvement in performance beyond what could be achieved with the default parameters. A key question was how best to optimize both the AlphaFold internal parameters and the two logistic regression parameters. One alternative would be to simultaneously fit both the AlphaFold parameters and the logistic regression parameters. Since AlphaFold has 100s of millions of parameters that collectively summarize information on the protein sequence-structure mapping, and we are fine-tuning on a very limited domain (peptide-MHC interactions), we were concerned about moving too far from the starting parameters and thereby losing general structural knowledge that could be relevant for new protein-peptide systems not seen during training. This desire to be conservative in the fine-tuning guided many of our decisions, from stopping the AlphaFold fine-tuning after just two epochs (i.e., each example seen twice), to this question of how to train the binder model. For example, if we started with randomly initialized logistic regression parameters, early training steps might perturb the internal AlphaFold parameters in unphysical directions. Thus, we decided to first fit the two logistic regression parameters in the context of the starting AlphaFold parameters, and then keep them fixed during the fine-tuning of the AlphaFold parameters. This also avoids 'mixing' of different parameter types during training (i.e., logistic regression parameters and AlphaFold internal parameters). To explore this question further, we re-ran model training several times with random initial logistic regression parameters and saw that AUROC values of the final models were comparable to or lower than the model described in the main text. We also tried initializing the logistic regression parameters to their fitted values and letting them vary during training, and we found that they remained essentially unchanged during fine-tuning of the AlphaFold parameters, and the final model performed equivalently to the one described above.

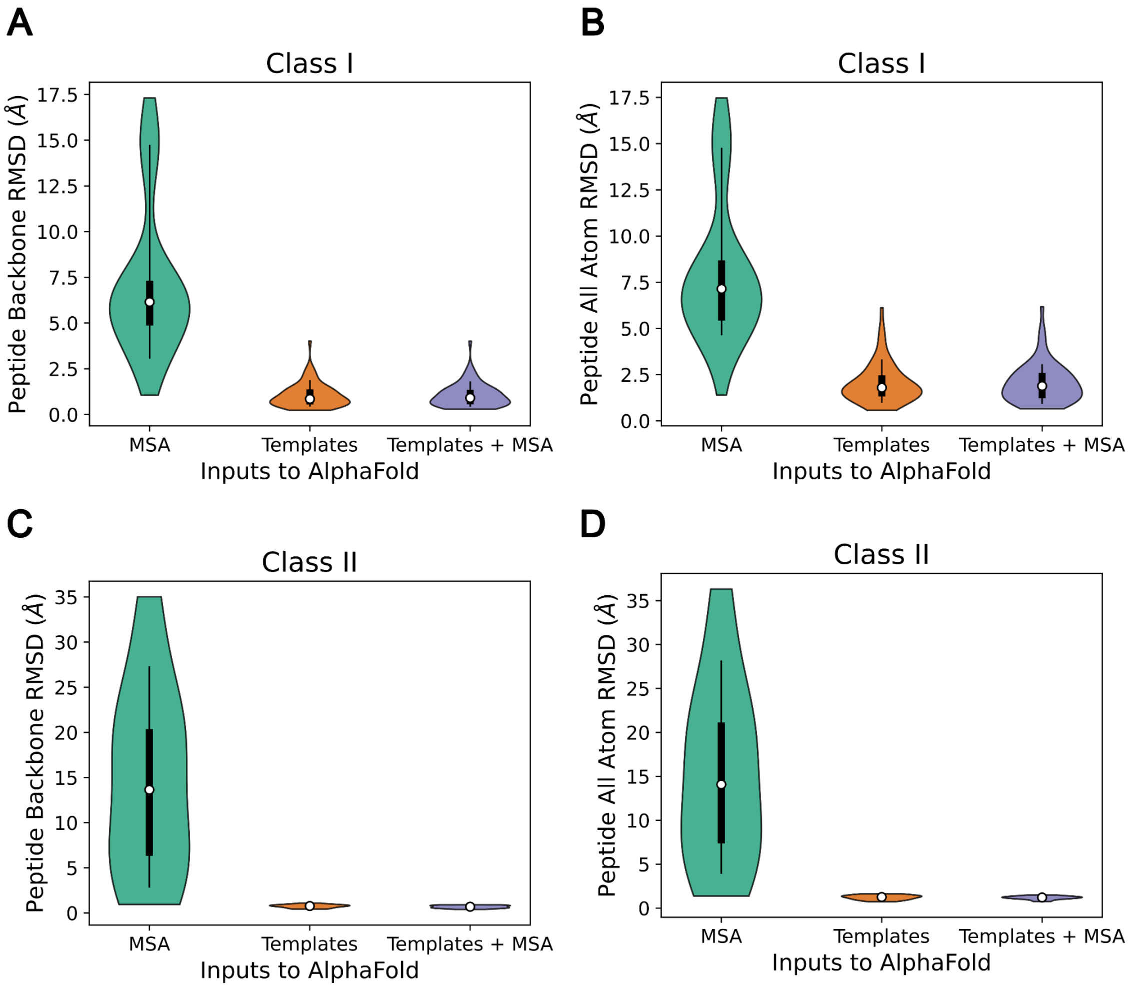


**Fig. S1. Comparison of peptide-MHC structure modeling quality of AlphaFold with different inputs.** Violin plots of peptide RMSD distribution of peptide-MHC models produced by AlphaFold with MSA, Templates, or MSA and Templates as inputs to the network. (A) peptide backbone RMSD for Class I, (B) peptide all atom RMSD for Class I (C) peptide backbone RMSD for Class II and (D) peptide all atom RMSD for Class II complexes. White circles represent the median, thick lines the interquartile range, and thin lines the range between 10th and 90th percentiles.

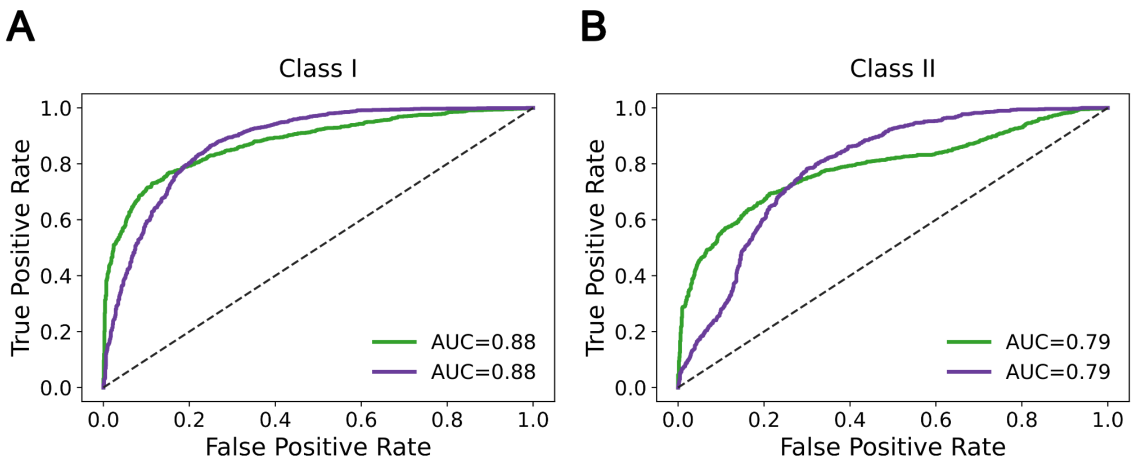


**Fig. S2. Peptide-MHC classification with AlphaFold structure accuracy metrics.** ROC curves comparing AlphaFold's confidence metrics in binder/non-binder peptide classification for (A) MHC Class I and (B) MHC Class II. Mean inter-chain PAE (green) and mean peptide pLDDT (purple).

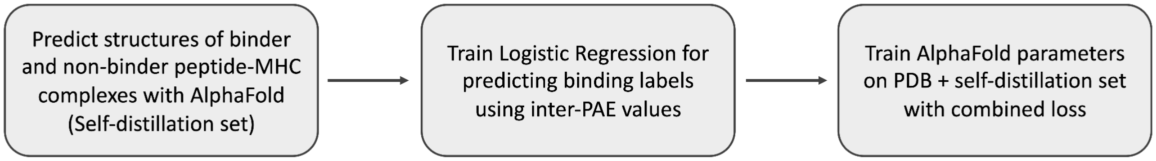


**Fig. S3. Flowchart of the combined structure prediction-classification fine-tuning on peptide-MHC dataset.** Overview of the steps regarding the combined fine-tuning procedure explained in the Methods section.

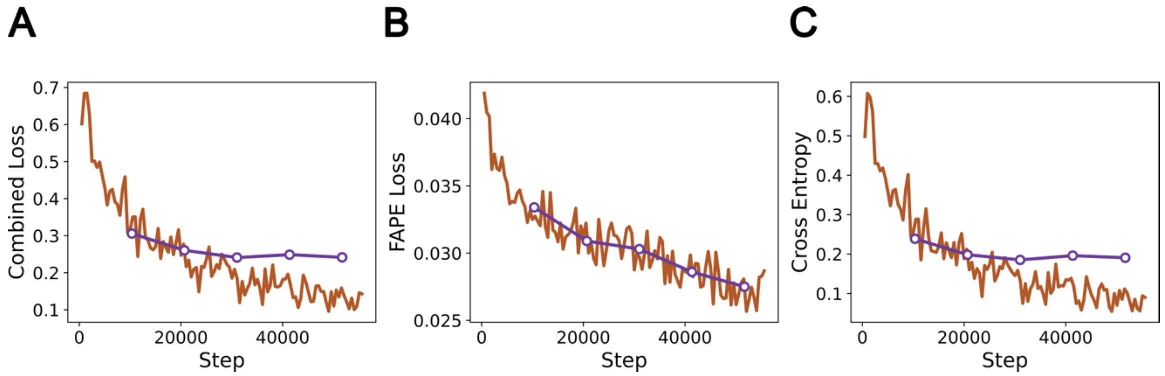


**Fig. S4. Loss progression curve during fine-tuning of AlphaFold on peptide-MHC structures. A-C.** Plots of (A) combined fine-tuning loss, (B) FAPE loss on structures (crystal structures and predicted self-distillation structures), and (C) cross entropy of peptide-MHC binding classification against training steps in the combined structural and classification fine-tuning of AlphaFold parameters on peptide-MHC data. Training loss (maroon) and validation loss (purple).

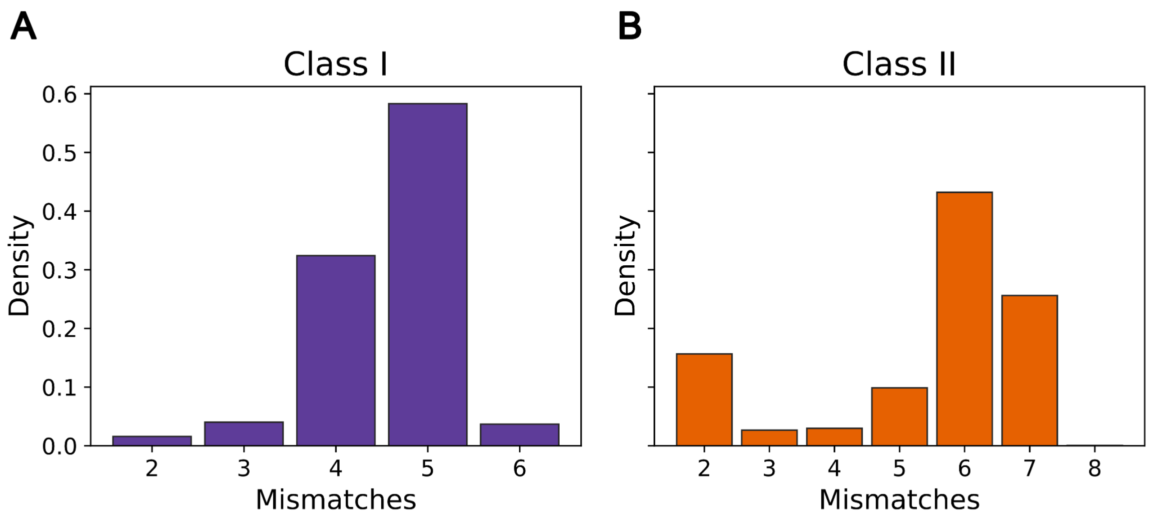


**Fig. S5. Histogram of peptide distance in validation set compared to train set.** Minimum number of mismatches of peptides in the validation set compared to any peptide in the training set for (A) MHC Class I and (B) MHC Class II.

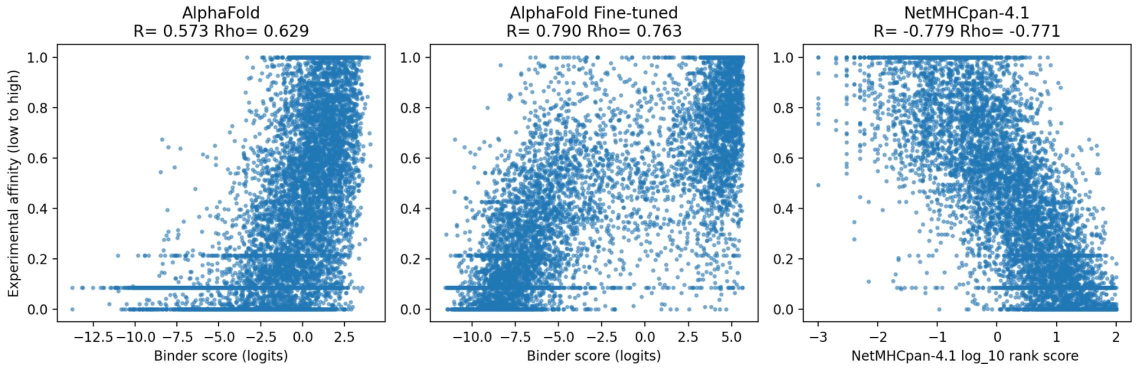


**Fig. S6. Correlation between predicted and experimental binding affinities for 9 residue peptides binding to the HLA allele A*02:01.** Predictions for AlphaFold are shown on the left, for the fine-tuned binder model in the middle, and for NetMHCpan-4.1 on the right. Experimental binding affinities were taken from the NetMHCpan binding affinity training set (1). Affinities in that set have been log-transformed and scaled so that a value of 0 corresponds to weak binding (>= 50 mM) and a value of 1 corresponds to strong binding (<= 1 nM). Only peptides that did not overlap with the binder fine-tuning training set for any HLA-A*02 allele were included in the analysis. Pearson linear correlation coefficients (R values) and Spearman rank correlations (rho values) are shown in the panel titles. The NetMHCpan-4.1 rank score was $\log_{10}$-transformed (the untransformed rank score showed a weaker Pearson correlation coefficient of R=-0.537).

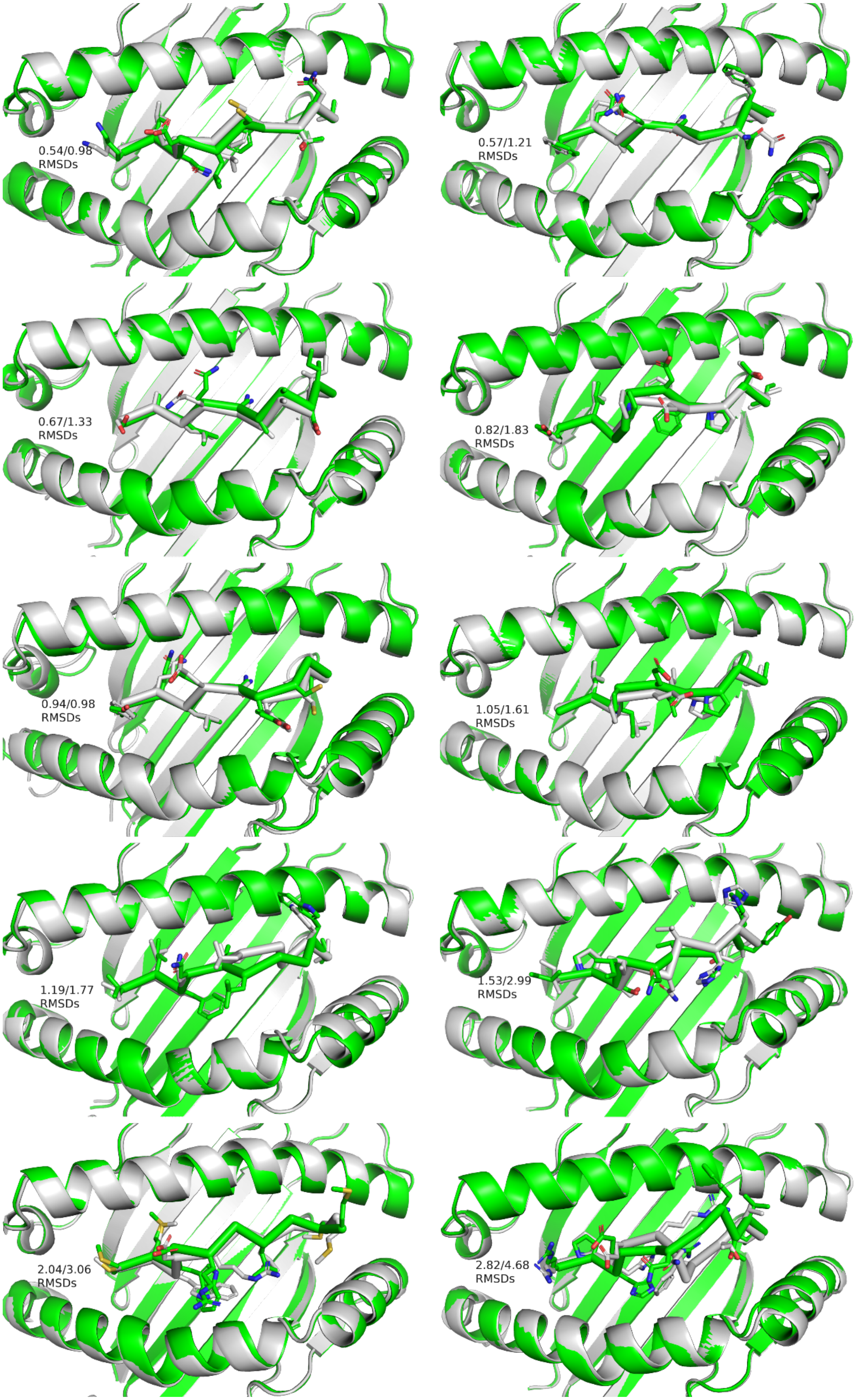
0.54/0.98
RMSDs
0.57/1.21
RMSDs
0.67/1.33
RMSDs
0.82/1.83
RMSDs
0.94/0.98
RMSDs
1.05/1.61
RMSDs
1.19/1.77
RMSDs
1.53/2.99
RMSDs
2.04/3.06
RMSDs
2.82/4.68
RMSDs

**Fig. S7. Representative class I peptide-MHC structural models generated by the fine-tuned binder model.** Target peptides have at least 2 mismatches to any binder peptide in the training set. Modeled structure is shown in green and native in gray. Backbone/all-atom RMSD values are shown to the left of the peptide, which is oriented with the N-terminus on the left. Representative structures were chosen uniformly along the backbone RMSD distribution (median 0.98 Å backbone and 1.83 Å all-atom RMSD).

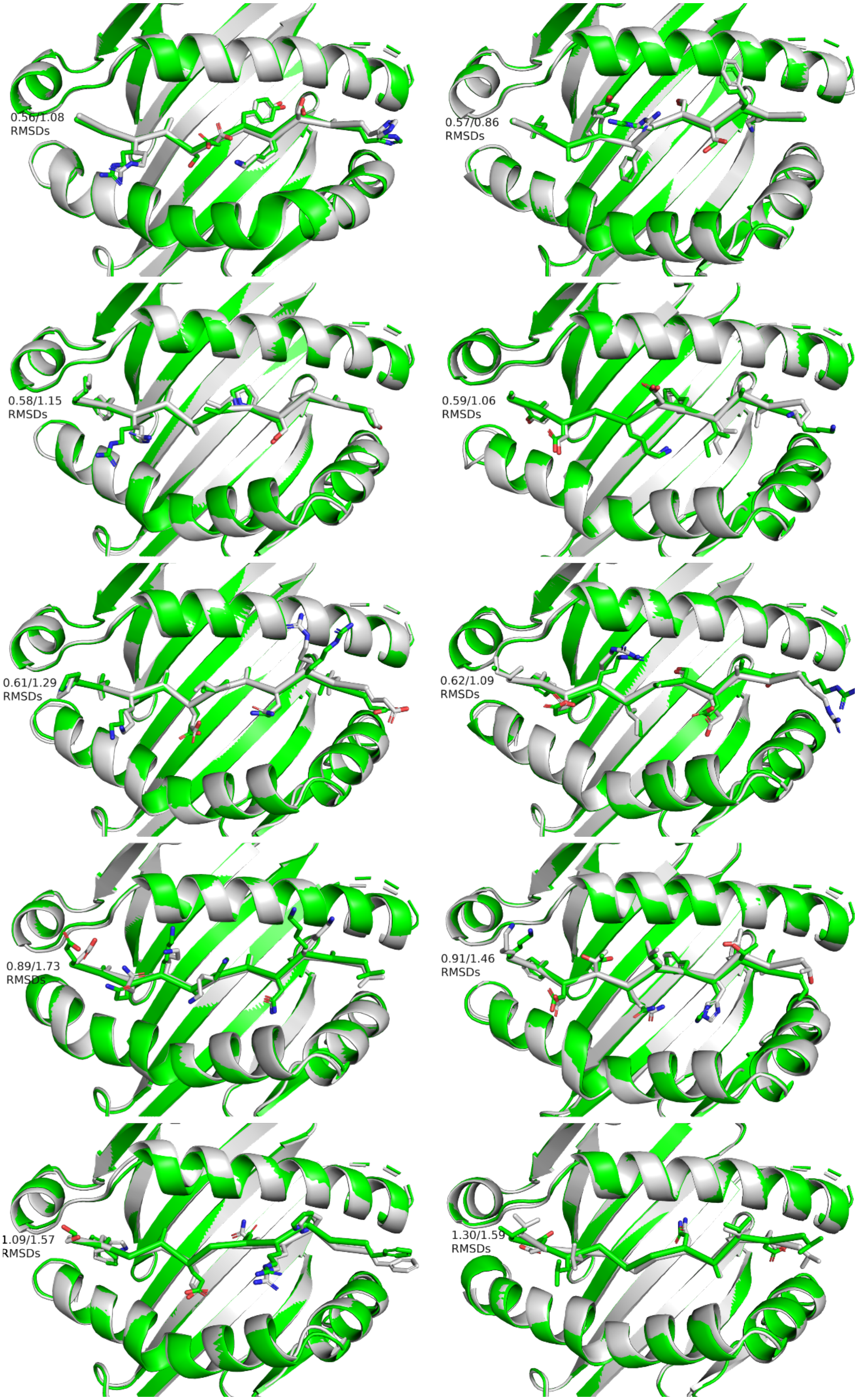
0.56/1.08
RMSDs
0.57/0.86
RMSDs
0.58/1.15
RMSDs
0.59/1.06
RMSDs
0.61/1.29
RMSDs
0.62/1.09
RMSDs
0.89/1.73
RMSDs
0.91/1.46
RMSDs
1.09/1.57
RMSDs
1.30/1.59
RMSDs

**Fig. S8. Representative class II peptide-MHC structural models generated by the fine-tuned binder model.** Target peptides have at least 2 mismatches to any binder peptide in the training set. Modeled structure is shown in green and native in gray. Backbone/all-atom RMSD values are shown to the left of the peptide, which is oriented with the N-terminus on the left. Representatives were chosen uniformly along the backbone RMSD distribution (median 0.61 Å backbone and 1.15 Å all-atom RMSD).

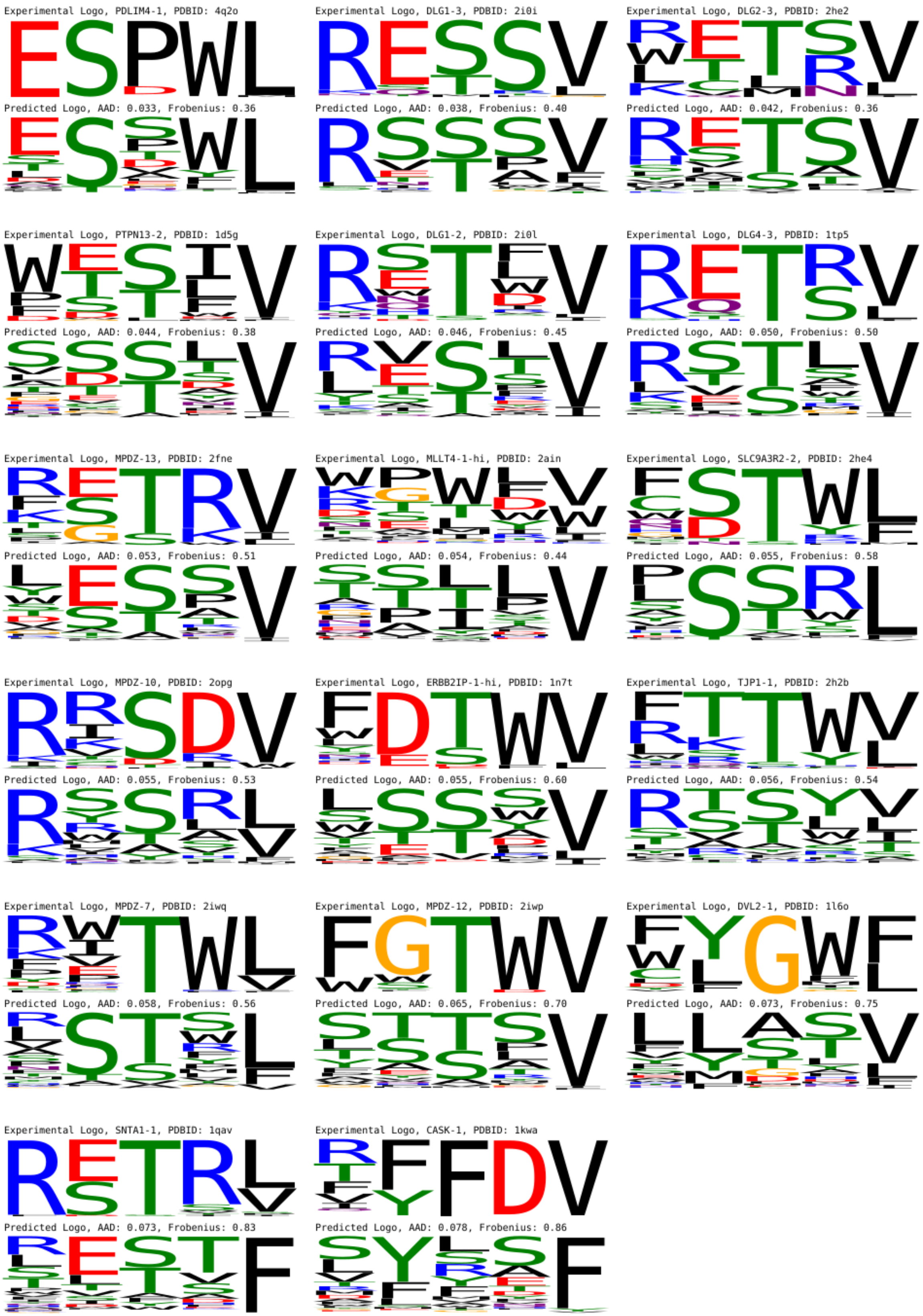


**Fig. S9. Predicted and experimental sequence logos for the 17 PDZ domains.** Predicted logos were built from the top 1% of random peptides ranked by the combined structure prediction-

classification model. Domains are ordered by increasing AAD between predicted and experimental logos.

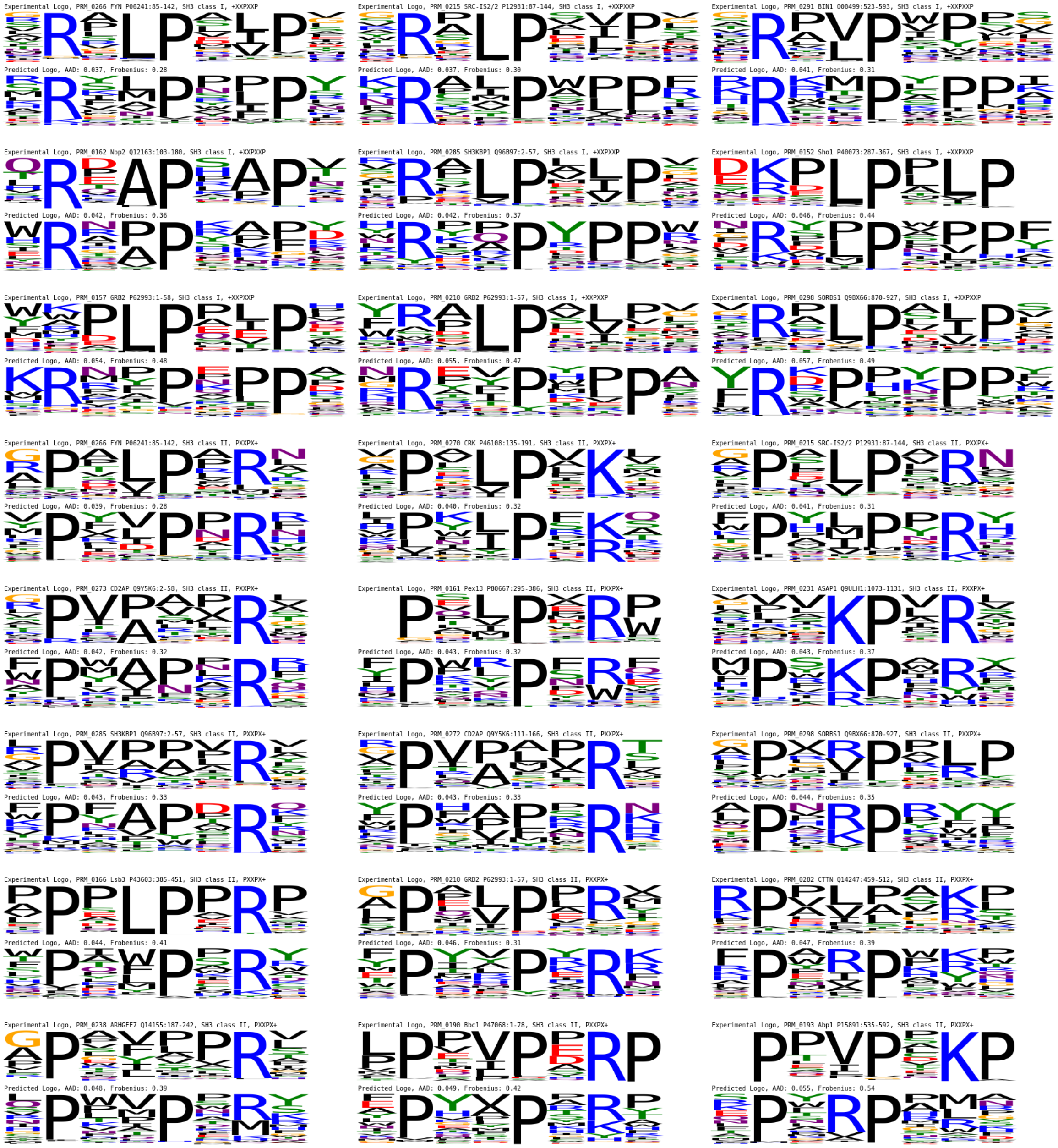

**Fig. S10. Predicted and experimental sequence logos for the 24 SH3 domains.** Predicted logos were built from the top 1% of random peptides ranked by the combined structure prediction-classification model. Domains are grouped by SH3 class and ordered within each class by increasing AAD between predicted and experimental logos.

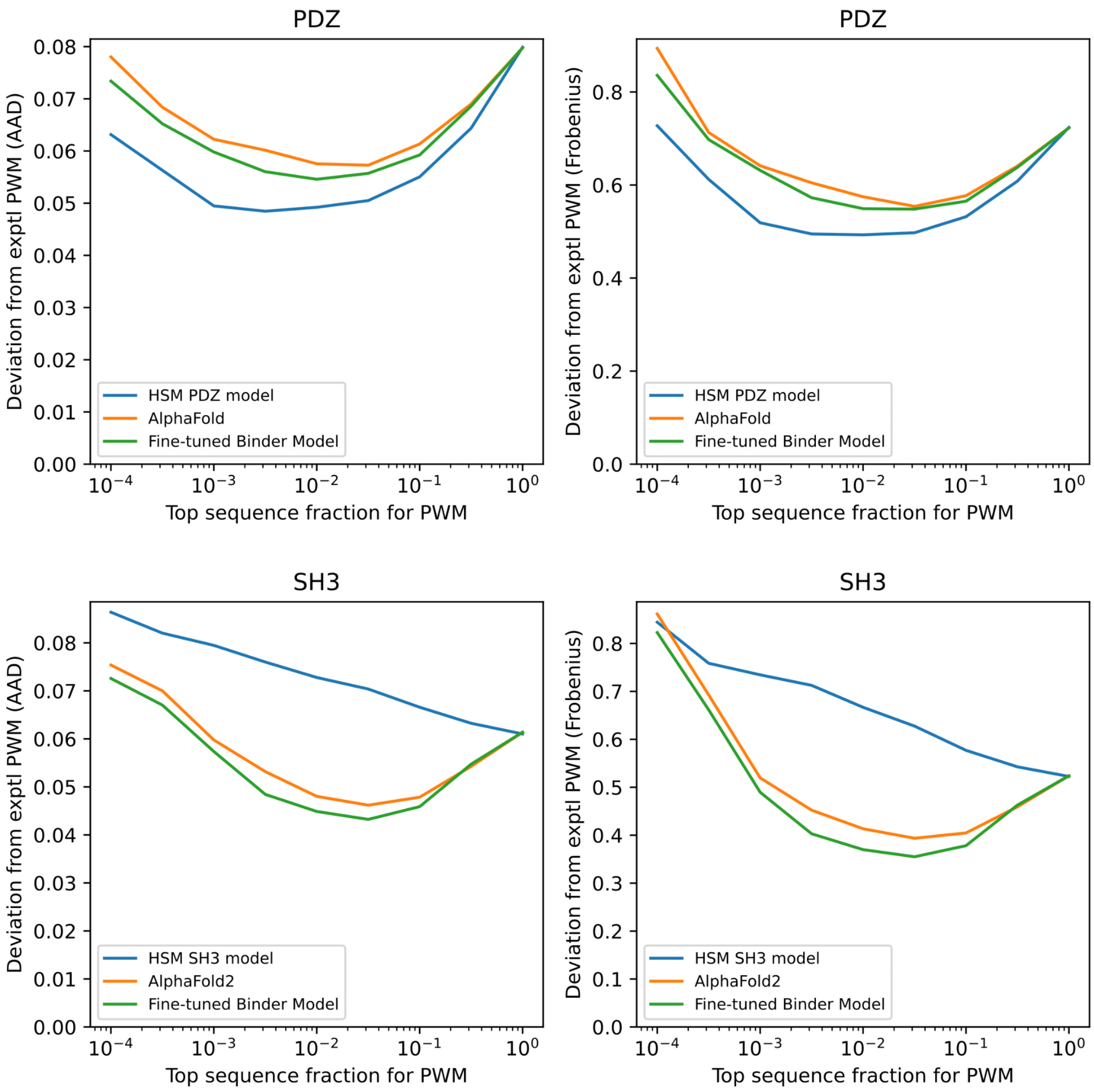


**Fig. S11. PWM prediction accuracy for structure-based models and for the HSM machine learning approach.** As described in the main text, twenty thousand random peptides were ranked with the structure-based approaches (standard AlphaFold with inter-PAE ranking and the fine-tuned binder model) and with the sequence-based machine learning approach hierarchical statistical mechanical modeling (HSM) (2). PWMs were generated from the top fraction of the ranked sequences as indicated on the x-axis, and these PWMs were compared with experimental PWMs using the AAD (left) and Frobenius (right) measures (see Methods). PDZ PWMs were aligned based on the position of the C-terminus; SH3 PWMs were aligned based on the PxxP motif (structure-based approaches) or by trying all registers and taking the one with minimal deviation (HSM). Pre-trained HSM PDZ and SH3 models and prediction scripts were downloaded from https://github.com/aqlaboratory/hsm. The HSM approach outperforms the structure-based models for PDZ domains, suggesting that further performance gains could be achieved by including PDZ binding data in the training set used for fine-tuning.

**Table S1. PDZ domain dataset**

| PDZ domain | Template[a] | Template peptide |
|---|---|---|
| CASK-1 | 1kwa_AB | VPSYREF |
| DLG1-2 | 2i0l_AC | RRETQV |
| DLG1-3 | 2i0i_AD | RRETQV |
| DLG2-3 | 2he2_AB | KIHETSV |
| DLG4-3 | 1tp5_AB | KKETWV |
| DVL2-1 | 1l6o_AD | LKLMTTV |
| ERBB2IP-1-hi | 1n7t_AB | TGWETWV |
| MLLT4-1-hi | 2ain_AB | LFSTEV |
| MPDZ-7 | 2iwq_AA | SIISTRL |
| MPDZ-10 | 2opg_AB | PYKSTRL |
| MPDZ-12 | 2iwp_BA | DVSETSV |
| MPDZ-13 | 2fne_BA | SSDETSV |
| PDLIM4-1 | 4q2o_AB | VESPWL |
| PTPN13-2 | 1d5g_AB | NEQVSAV |
| SLC9A3R2-2 | 2he4_AA | VGPSTRL |
| SNTA1-1 | 1qav_AB | THLETTF |
| TJP1-1 | 2h2b_AA | WRRTTYL |

[a]PDB ID followed by PDZ and peptide chain identifiers

**Table S2. SH3 domain dataset**

| PRM-DB identifier[a] | SH3 class | Protein name | Uniprot identifier | SH3 template[b] | Peptide templates[c] |
|---|---|---|---|---|---|
| PRM_0152 | 1 | Sho1 | P40073:287-367 | 2vkn_A | 2vkn_AC,3ua7_AE,4rtz_AB,5sxp_BF |
| PRM_0157 | 1 | GRB2 | P62993:1-58 | 1gbq_A | 5sxp_BF,1io6_AB,2bz8_AC,3ua7_AE |
| PRM_0161 | 2 | Pex13 | P80667:295-386 | 1n5z_A | 1n5z_AP,1gbq_AB,1gbq_AB,5ul6_AM |
| PRM_0162 | 1 | Nbp2 | Q12163:103-180 | 2lcs_A | 2lcs_AB,2bz8_AC,1io6_AB,4rtz_AB |
| PRM_0166 | 2 | Lsb3 | P43603:385-451 | 1ssh_A | 1ssh_AB,3ua7_DF,2d1x_DQ,5ul6_AM |
| PRM_0190 | 2 | Bbc1 | P47068:1-78 | 1zuk_B | 2rqu_AB,4ln2_AB,2d1x_DQ,2rpn_AB |
| PRM_0193 | 2 | Abp1 | P15891:535-592 | 2rpn_A | 2rpn_AB,2d1x_DQ,4wci_EF,2rqu_AB |
| PRM_0210 | 1 | GRB2 | P62993:1-57 | 1gbq_A | 5sxp_BF,1io6_AB,2bz8_AC,3ua7_AE |
| PRM_0210 | 2 | GRB2 | P62993:1-57 | 1gbq_A | 1gbq_AB,1gbq_AB,2d1x_DQ,5xhz_AC |
| PRM_0215 | 1 | SRC-IS2/2 | P12931:87-144 | 4rtz_A | 4rtz_AB,3ua7_AE,5sxp_BF,1io6_AB |
| PRM_0215 | 2 | SRC-IS2/2 | P12931:87-144 | 4rty_A | 4rty_AB,3ua7_DF,5ul6_AM,4f14_AB |
| PRM_0231 | 2 | ASAP1 | Q9ULH1:1073-1131 | 2rqu_A | 2rqu_AB,5xhz_AC,2rpn_AB,2d1x_DQ |
| PRM_0238 | 2 | ARHGEF7 | Q14155:187-242 | 5sxp_B | 5xhz_AC,3u23_AB,2d1x_DQ,3ua7_DF |
| PRM_0266 | 1 | FYN | P06241:85-142 | 3ua7_A | 3ua7_AE,4rtz_AB,5sxp_BF,1io6_AB |
| PRM_0266 | 2 | FYN | P06241:85-142 | 3ua7_D | 3ua7_DF,4rty_AB,5ul6_AM,1gbq_AB |
| PRM_0270 | 2 | CRK | P46108:135-191 | 5ul6_A | 5ul6_AM,4rty_AB,1gbq_AB,1gbq_AB |
| PRM_0272 | 2 | CD2AP | Q9Y5K6:111-166 | 3u23_A | 3u23_AB,5xhz_AC,4wci_EF,2d1x_DQ |
| PRM_0273 | 2 | CD2AP | Q9Y5K6:2-58 | 4wci_E | 4wci_EF,5xhz_AC,3u23_AB,2rpn_AB |
| PRM_0282 | 2 | CTTN | Q14247:459-512 | 2d1x_D | 2d1x_DQ,4f14_AB,2rpn_AB,1gbq_AB |
| PRM_0285 | 1 | SH3KBP1 | Q96B97:2-57 | 2bz8_A | 2bz8_AC,5sxp_BF,1io6_AB,3ua7_AE |
| PRM_0285 | 2 | SH3KBP1 | Q96B97:2-57 | 2bz8_A | 4wci_EF,2d1x_DQ,5xhz_AC,3u23_AB |
| PRM_0291 | 1 | BIN1 | O00499:523-593 | 5i22_A | 5sxp_BF,2bz8_AC,1io6_AB,3ua7_AE |
| PRM_0298 | 1 | SORBS1 | Q9BX66:870-927 | 4ln2_A | 5sxp_BF,2bz8_AC,1io6_AB,3ua7_AE |
| PRM_0298 | 2 | SORBS1 | Q9BX66:870-927 | 4ln2_A | 4ln2_AB,1gbq_AB,1gbq_AB,2rqu_AB |

[a]Internal identifier used in the PRM-DB database (http://prm-db.org/)

[b]PDB and chain identifier of SH3 domain modeling template

[c]PDB identifier followed by SH3 and peptide chain identifiers of peptide modeling templates

**SI References**


1. B. Reynisson, B. Alvarez, S. Paul, B. Peters, M. Nielsen, NetMHCpan-4.1 and NetMHCIIpan-4.0: improved predictions of MHC antigen presentation by concurrent motif deconvolution and integration of MS MHC eluted ligand data. Nucleic Acids Res. 48, W449–W454 (2020).
2. J. M. Cunningham, G. Koytiger, P. K. Sorger, M. AlQuraishi, Biophysical prediction of protein-peptide interactions and signaling networks using machine learning. Nat. Methods 17, 175–183 (2020).
